# Fecal Bacterial Community of Allopatric Przewalski’s Gazelles and Their Sympatric Relatives

**DOI:** 10.3389/fmicb.2021.737042

**Published:** 2021-09-24

**Authors:** Ruoshuang Liu, Jianbin Shi, Susanne Shultz, Dongsheng Guo, Dingzhen Liu

**Affiliations:** ^1^School of Environment, Beijing Normal University, Beijing, China; ^2^Department of Earth and Environmental Sciences, University of Manchester, Manchester, United Kingdom; ^3^Key Laboratory of Biodiversity Sciences and Ecological Engineering, College of Life Sciences, Beijing Normal University, Beijing, China

**Keywords:** Przewalski’s gazelle, *Procapra przewalskii*, 16S rRNA gene, gut microbiome, ruminant, diet, Tibetan gazelle

## Abstract

Mammal gastrointestinal tracts harbor diverse bacterial communities that play important roles in digestion, development, behavior, and immune function. Although, there is an increasing understanding of the factors that affect microbial community composition in laboratory populations, the impact of environment and host community composition on microbiomes in wild populations is less understood. Given that the composition of bacterial communities can be shaped by ecological factors, particularly exposure to the microbiome of other individuals, inter-specific interactions should impact on microbiome community composition. Here, we evaluated inter-population and inter-specific similarity in the fecal microbiota of Przewalski’s gazelle (*Procapra przewalskii*), an endangered endemic ruminant around Qinghai Lake in China. We compared the fecal bacterial communities of three Przewalski’s gazelle populations, with those of two sympatric ruminants, Tibetan gazelle (*Procapra picticaudata*) and Tibetan sheep (*Ovis aries*). The fecal bacterial community richness (Chao1, ACE) did not vary across the three Przewalski’s gazelle populations, nor did the composition vary between species. In contrast, the managed Przewalski’s gazelle population had higher bacterial diversity (Shannon and Simpson) and was more similar to its sympatric Tibetan sheep in beta diversity than the wild Przewalski’s gazelle populations. These results suggest that ecological factors like host community composition or diet affect Przewalski’s gazelle’s gastrointestinal bacterial community. The role of bacterial community composition in maintaining gastrointestinal health should be assessed to improve conservation management of endangered Przewalski’s gazelle. More broadly, captive breeding and reintroduction efforts may be impeded, where captive management results in dysbiosis and introduction of pathogenic bacteria. In free ranging populations, where wildlife and livestock co-occur, infection by domestic pathogens and diseases may be an underappreciated threat to wild animals.

## Introduction

Animals harbor microorganism communities in a number of habitats (West et al., 2019) that play key roles in host biology (Mueller and Sachs, 2015; Sun et al., 2018). The gastrointestinal tract contains a complex ecological system of diverse microbial communities, including bacteria, archaea, viruses, and eukaryotic microbes such as fungi and protozoa (McFall-Ngai et al., 2013; Underhill and Lliev, 2014).

A growing number of studies have shown the biological functions of gut microbiome for cellulose digestion, nutrient absorption, energy harvest, vitamin synthesis, metabolic disorders, development, immune function, behavior, and protection against pathogens (Hooper and Gordon, 2001; Dale and Moran, 2006; Round and Mazmanian, 2009; Archie and Theis, 2011; Wu et al., 2011; Ezenwa et al., 2012; Greenblum et al., 2012; Hooper et al., 2012; Abt and Pamer, 2014; Sampson and Mazmanian, 2015). The mammalian gastrointestinal microbiome is dominated by Firmicutes and Bacteroidetes, which contain many anaerobic fermentative bacterial species. These bacteria digest and ferment plant structural carbohydrates into available products like short-chain fatty acids (SCFAs, predominantly acetate, butyrate, and propionate), which are absorbed by the host (Amato et al., 2016; Lan and Yang, 2019; Sun et al., 2020). Herbivores and particularly ruminants depend on the microbial fermentation products and other metabolites such as vitamins and high-quality proteins for more than two-thirds of their daily energetic requirements (Morgavi et al., 2013; Furman et al., 2020). This long-time co-evolved holobiont (the host and its symbiotic microbiota) also has adaptive plasticity to external environments. For example, high altitude yaks (*Bos grunniens*) and Tibetan sheep (*Ovis aries*) yield significantly less methane and more volatile fatty acids (VFAs) than their low-altitude relatives, e.g., cattle (*Bos taurus*) and sheep (*O. aries*) as a result of changes in rumen microbiome structure and composition (Zhang et al., 2016). This convergent adaption of yaks and Tibetan sheep increases their feeding efficiency to cope with a harsh high-altitude environment (Zhang et al., 2016).

The relationship between hosts and microbiota is important for conservation and species management because changes in the gastrointestinal microbiome affect host nutrition and health (Amato et al., 2013; Zhu et al., 2021). Habitat degradation, community composition, and captivity impact on the gut microbiome of threatened species (West et al., 2019). Loss of plant taxa in degraded habitats, or simple and homogenous low-fiber diets in management programs, could lead to the loss of key plant-digesting microbiota and reduce the functional capacity of the host gut microbiome (Borbón-García et al., 2017; West et al., 2019). Decreased gut microbiota diversity is thought to negatively impact host survivorship by both reducing its ability to process plant compounds and is associated with increased gastrointestinal tract inflammation (Hale et al., 2019; West et al., 2019). Furthermore, dysbiosis of the gut microbiota can result in a decrease in beneficial microbes, an increase in pathogenic microbes, or an altered metabolic environment in the gut, potentially reducing microbiome function and yielding a less efficient and resistant microbiome that is susceptible to infection and disease (Gilbert et al., 2018; Hale et al., 2019; West et al., 2019). These changes to the gut microbiome could be a crucial but underappreciated risk to threatened animals both in the wild and in captivity.

The composition and abundance of gut microbiota are shaped by heritable factors such as host genetics, evolutionary history, and vertical generation-to-generation transmission (Ochman et al., 2010; Vaishampayan et al., 2010; Goodrich et al., 2014; Moeller et al., 2014), as well as ecological factors such as host diet and geography (Ley et al., 2008; Muegge et al., 2011; Yatsunenko et al., 2012; Moeller et al., 2013; David et al., 2014; Song et al., 2017; Zhu et al., 2021). The microbial composition of Bighorn sheep (*Ovis canadensis nelsoni*) is mediated by host genetic heterozygosity as well as geographic proximity (Couch et al., 2020). The gut microbiota of captive Cercopithecinae and Colobinae primates can be clustered strongly by subfamily, but weakly by species, reflecting adaptations associated with their respective diets in the context of host phylogeny (Huan et al., 2020). In herbivorous animals like American bison (*Bison bison*; Bergmann et al., 2015), African buffalo (*Syncerus caffer*; Couch et al., 2021a), Tibetan macaque (*Macaca thibetana*; Sun et al., 2018), Western lowland gorillas (*Gorilla gorilla*; Gomez et al., 2015; Hicks et al., 2018), Chimpanzees (*Pan troglodytes*; Hicks et al., 2018), and Black howler monkeys (*Alouatta pigra*; Amato et al., 2015), symbiotic gut microbiota varies due to seasonal changes in their food resources. Such variation is believed to help the host animals improve energy intake efficiency and adapt to the changing environment. Spatial overlap between herbivorous hosts also contributes to microbiome community similarity: individuals either in the same social group or with overlapping home ranges have more similar microbiome communities than individuals that do not spatially overlap (Antwis et al., 2018). Additionally, sympatric species share microbiota (Moeller et al., 2013; Perofsky et al., 2019), suggesting that host community composition can be an important driver of microbiome diversity and composition.

The Przewalski’s gazelle (*Procapra przewalskii*) is a group-living Bovid that once occupied areas from the Qinghai Province to Inner Mongolia. It has undergone extensive range contraction and population collapse and is now limited to small isolated sub-populations around Qinghai Lake in China and listed as endangered (EN) by the IUCN Red List [Li et al., 2010; International Union for Conservation of Nature (IUCN) Species Survival Commission (SSC) Antelope Specialist Group, 2016]. The Tibetan gazelle (*Procapra picticaudata*) is a congener of Przewalski’s gazelle and has high overlap in diet (Li et al., 2008), but it is one of the most geographically widespread ungulates on the Qinghai-Tibetan Plateau. It also occurs in fragmented habitat patches and mixed groups of Przewalski’s gazelle and Tibetan gazelle are common on the plateau during summer and winter (Li et al., 2008, 2010). In addition, livestock such as Tibetan sheep occur on the plateau and share many food resources with the sympatric wild ungulates (Li et al., 2008). Thus, there are complex spatial dynamics between Przewalski’s gazelle sub-populations as well as between Przewalski’s gazelles and other grazers on the plateau.

As foregut fermenters, Przewalski’s gazelles have long gut retention time to allow efficient microbial fermentation to digest the complex carbohydrates present in plants, such as celluloses and resistant starches (Ley et al., 2008). Ruminant gastrointestinal tracts are generally inhabited by a high density and diversity of microbiota with complex interactions (Brulc et al., 2009). Przewalski’s gazelle and several other sympatric ruminants have adapted to live in the extreme high-altitude environment of the Qinghai-Tibetan Plateau (Zhang et al., 2016), but little is currently known about their microbiota. Given the opportunity for high altitude microbiome adaptation and the structured spatial interactions between herbivore species on the plateau, exploring the structure and function of the gastrointestinal microbiome of Przewalski’s and Tibetan gazelles and other sympatric ruminants provides an excellent system for understanding the dynamics of microbiome communities within and between species.

Here, we examined and compared the gastrointestinal microbiota of wild and managed populations of Przewalski’s gazelle with two sympatric ruminants, Tibetan gazelle and Tibetan sheep, using high-throughput Illumina sequencing based on the 16S ribosomal RNA gene. An effective method to disentangle the effects of heritable vs. ecological factors on the gut microbiota is to compare sympatric (i.e., habitat overlapped) and allopatric (i.e., geographically separated) host populations (Moeller et al., 2013). Microbiomes vary across the digestive track depending on nutrient availability, pH, and digestive enzymes, with a characteristic foregut and hindgut microbiome in ruminants (Holman and Gzyl, 2019). However, population differences are consistent between foregut and hindgut microbial composition such that the fecal microbiome can be used as a proxy for gastrointestinal microbiota in wildlife studies (Liu et al., 2018). In the current study, three geographically separated Przewalski’s gazelle populations were chosen to understand population differences in the structure and function of the gut microbiota: one sympatric with Tibetan gazelle, one with Tibetan sheep, and the remaining one without sympatric ruminants. We characterized the fecal bacterial community of the three populations of Przewalski’s gazelles, sympatric Tibetan gazelle, and Tibetan sheep, to address the question of whether the fecal bacterial community of Przewalski’s gazelle was better predicted by phylogeny or ecological characteristics. We hypothesized that: (1) the fecal bacterial community was primarily determines by heritable factors, the microbiota of wild and managed Przewalski’s gazelle populations would be more similar compared to sympatric other ruminants; and (2) if ecological factors determined the fecal bacterial community of Przewalski’s gazelle, bacterial community composition should be more similar to the sympatric ruminants than to other populations of Przewalski’s gazelles.

## Materials and Methods

### Ethical Statement

There was no direct contact with animals in this study, and no animals were manipulated to collect the data. All the fecal samples and behavioral data were collected in compliance with Law of the People’s Republic of China on the Protection of Wildlife (The National People’s Congress of the People’s Republic of China, 2016).

### Sample Collection

**Figure 1:**
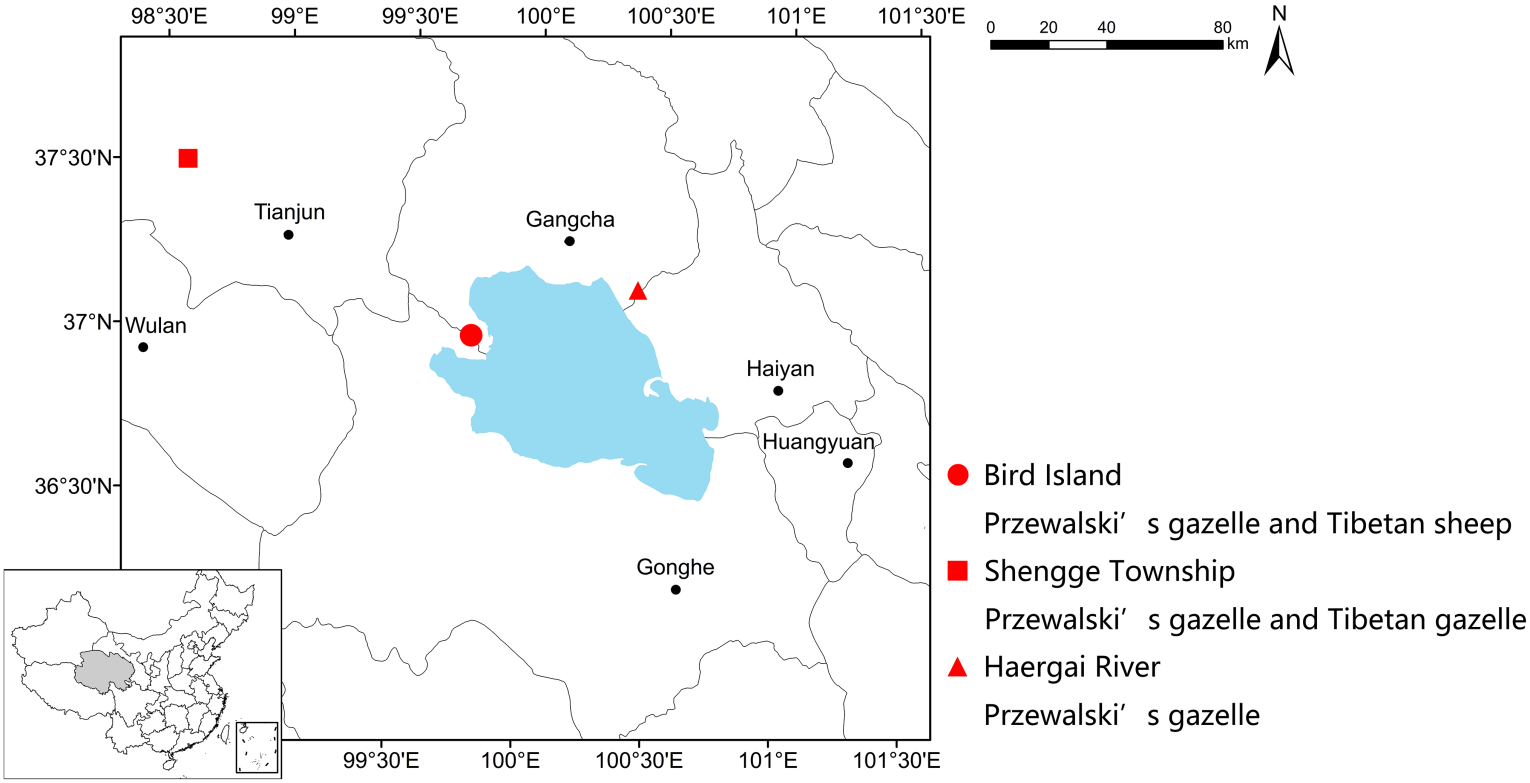
Geographic locations of sample collection sites. This study included one managed Przewalski’s gazelle population sympatric with Tibetan sheep (Bird Island, red dot), one wild Przewalski’s gazelle population sympatric with Tibetan gazelle (Shengge Township, red square), and one wild Przewalski’s gazelle population with no sympatric ruminants (Haergai River, red triangle).

Fecal samples were collected in December 2016 from three study sites, i.e., the Buha River Valley in the Shengge Township (98°31'49.57''E, 37°29'07.02''N, 3,685ma.s.l.), the downstream Haergai River (100°27'25.74''E, 37°11'50.34''N, 3,254 m a.s.l.), and Bird Island Protection Station (99°51'22.77''E, 36°59'13.35''N, 3,195 m a.s.l.; Figure 1). The climate type is plateau continental (approximate temperature range from −20 to 20°C), which is cold and dry, with strong solar radiation and wind. The warmest month is July and the coldest month is January. More than 60% of the ~300mm of annual precipitation falls during the wet humid summer (June–September), while most of the rest falls during the arid winter (November–February). The vegetation in Shengge is mainly alpine meadow, alpine steppe, and alpine desert steppe, dominated by *Kobresia* spp., *Carex supina*, *Artemisia* spp., *Poa* spp., and *Stipa purpurea*; while Haergai and Bird Island is mainly alpine meadow and temperate steppe, dominated by *Achnatherun splendens*, *Stipa* spp., *Agropyron cristatum*, *Leymus secalinus*, and *Orinus kokonorica* (Qinghai Provincial Grassland Station, 2013). Although, there is diet overlap between all three species, Przewalski gazelle diet primarily contains a mixture of by legumes (Leguminosae), grasses (Gramineae), and daisies (Compositae), Tibetan gazelle diet has a higher prevalence of legumes and sheep diet is more grass (Gramineae and Cyperaceae) dominated (Liu and Jiang, 2004; Li et al., 2008). One population of approximately 80 Przewalski’s gazelles and one population of about 60 Tibetan gazelles were in the upstream Buha River valley, and they nonrandomly formed mixed species groups. The downstream Haergai River was home to over 600 Przewalski’s gazelles. There was a managed Przewalski’s gazelle population of 72 individuals freely ranging with approximately 20 Tibetan sheep in a 0.81km^2^ enclosure at the Bird Island Protection Station. The enclosure contains natural habitats including alpine steppe and a section of a river and surrounded by a 2m-high wire-netting fence with barbed wire on the top to prevent gazelles from escaping or entering. One ton of *Arrhenatherum elatius* hay was supplementally supplied to this managed population each month from December to March of the next year.

We provided information on study animals and their populations (Table 1) for clarity and easy reading.

**Table 1:** Information on study animals and their populations.

Species	Population	Location	Short name	Note
Przewalski’s gazelle	Wild	Shengge Township	PG-S	Sympatric Tibetan gazelle (TG-S)
	Wild	Haergai River	PG-H	
	Managed/Enclosure	Bird Island	PG-B	Sympatric Tibetan sheep (TS-B)
Tibetan gazelle	Wild	Shengge Township	TG-S	
Tibetan sheep	Managed/Enclosure	Bird Island	TS-B	

Although, Przewalski’s gazelles and Tibetan gazelles formed mixed-species associations, we collected all samples from single species groups. We used a telescope to observe defecations, recorded the place and picked up the fresh feces immediately after the animals moved away. This allowed us to determine easily whether a fecal sample collected belonged to Przewalski’s gazelle or Tibetan gazelle. Most of the sampled individuals were anonymous adult females. We used sterilized tubes to collect pellets from inside of the fecal pile, immediately after defecation to avoid contact with soil or other pollution sources. Samples were labeled and transported in an ice box, and then frozen at −20°C within 2h after defecation. We collected 26 Przewalski’s gazelle fecal samples (six from PG-S, 12 from PG-H, and eight from PG-B), 11 Tibetan gazelle fecal samples from TG-S and 13 Tibetan sheep fecal samples from TS-B. All samples at the same site were collected on the same day in order to avoid potential temporal variation in environmental factors impacting on the bacterial composition.

### DNA Extraction and 16S rRNA Gene Sequencing

Fecal samples were processed in the laboratory during March 2017. Microbial DNA was extracted from frozen fecal samples using the FastDNA SPIN Kit for Soil (MP Biomedicals, Santa Ana, CA, United States) according to manufacturer’s protocols. The final DNA concentration and purification were determined by NanoDrop 2000 UV-vis spectrophotometer (Thermo Scientific, Wilmington, United States), and the DNA integrity was examined with 1% agarose gel electrophoresis.

The V3–V4 hypervariable regions of the bacteria 16S ribosomal RNA gene were amplified using primers 338F (5'-ACTCCTACGGGAGGCAGCAG-3') and 806R (5'-GGACTACHVGGGTWTCTAAT-3') by thermocycler PCR system (GeneAmp 9700, ABI, United States). We conducted the PCR reactions as follows: 3min of denaturation at 95°C, 27cycles of 30s at 95°C, 30s for annealing at 55°C, and 45s for elongation at 72°C, and a final extension at 72°C for 10min. For PCR assays, 4μl of 5×Fast Pfu Buffer, 2μl of 2.5mM dNTPs, 0.8μl of each primer (5μΜ), 0.4μl of FastPfu Polymerase (TransGen, China), 0.2μl of BSA, 10ng of template DNA, and ddH_2_O were mixed to 20μl in total. We then extracted the resulted PCR products from a 2% agarose gel, purified them further using the AxyPrep DNA Gel Extraction Kit (Axygen Biosciences, Union City, CA, United States), and quantified them using QuantiFluor™-ST (Promega, United States) according to the manufacturer’s protocol. Finally, we pooled the purified amplicons in equimolar and paired-end sequenced (2×300bp) on an Illumina MiSeq platform (Illumina, San Diego, United States) according to the standard protocols by Majorbio Bio-Pharm Technology Co. Ltd. (Shanghai, China).

### Bioinformatics and Statistical Analysis

The raw sequencing data were analyzed on the free online platform of Majorbio I-Sanger Cloud Platform (Majorbio, China).^1^ Raw fastq files were demultiplexed, quality-filtered with Trimmomatic and merged with FLASH (version 1.2.11) according to the following criteria: (1) The 300bp reads were truncated at any site receiving an average quality score of <20 over a 50bp sliding window. The truncated reads shorter than 50bp and reads containing ambiguous characters were discarded; (2) Only overlapping sequences longer than 10bp were assembled according to their overlapped sequence. The maximum mismatch ratio of overlap region is 0.2. Reads that could not be assembled were discarded.

Operational taxonomic units (OTUs) were clustered with 97% similarity cutoff using UPARSE (version 7.1),^2^ and chimeric sequences were identified and removed using UCHIME (USEARCH, version 9.2). The taxonomy of each 16S rRNA gene sequence was analyzed by RDP Classifier Bayesian algorithm (version 2.2)^3^ against the SILVA (SSU128, accessed in May 2018) 16S rRNA database with a confidence threshold of 70%. We did not rarify or sub-sample our data as this can result in lost data and overdispersion leading to inflated Type I and Type 2 errors (McMurdie and Holmes, 2014).

Operational taxonomic unit rarefaction curves and sequencing depth index (Good’s coverage) were generated for each sample. Alpha-diversity indices, including community richness parameters (Chao1, ACE), community diversity parameters (Shannon, Simpson), were calculated with Mothur (version v.1.30.1).^4^ We used Kolmogorow-Smironov test to find the normality of these parameters, and all of them were normal distribution (Shannon: *p*=0.872, Simpson: *p*=0.11, ACE: *p*=0.67, Chao1: *p*=0.873). Thus, the Student’s *t*-test was used to analyze the diversity differences of populations. A general linear model was conducted to detect the effects of species and community compositions on bacterial diversity indices. We used unweighted UniFrac distances to measure beta diversity (inter-sample diversity) of the bacterial community, to build gut microbiota trees (Qiime 2 by UPGMA hierarchical clustering; Bolyen et al., 2019) and for the principal coordinate analysis (PCoA). Meanwhile, we performed the analysis of similarities (ANOSIM, analysis of ranked dissimilarities between two or more groups of sampling units) based on OTU compositions to analyze the differentiation in the bacterial community in fecal samples from different populations, and paired ANOSIM analysis to find out the similarities between each two populations (Clarke, 1993). To identify taxon differences between the species and populations, a Venn diagram was implemented using the R package VennDiagram (version 1.6.20)^5^ to show unique and shared taxa (Song et al., 2017). We used an ANOVA based on community richness to find the different bacterial taxa between populations, with false discovery rate (FDR) approach to adjust value of *p* in multiple tests, followed by the Scheffe test as a *post hoc* analysis. Subsequently, we conducted linear discriminant analysis effect size (LEfSe) to identify OTUs differentially represented between the five populations of ruminants, which takes into account both statistical significance and biological relevance (Segata et al., 2011). Threshold on the logarithmic LDA score was set at two for discriminative features, and one-against-all strategy was adopted for multi-class analyses. All statistical analyses were two sided with the significance level at 0.05.

The datasets analyzed in this study were submitted to NCBI Sequence Read Archive (SRA) under the accession number PRJNA684634.

## Results

### Bacterial Community Composition

**Figure 2:**
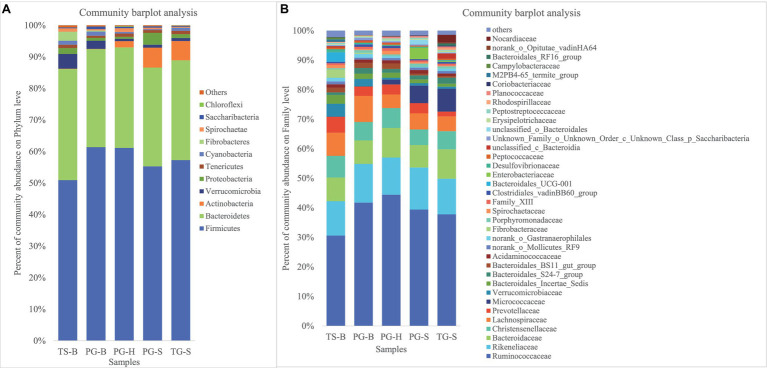
**(A)** Fecal bacterial community at the phylum levels. Phyla with less than 1% abundance were merged into others. **(B)** Fecal bacterial community at the family levels. Family with less than 1% abundance were merged into others. TS-B, Tibetan sheep on Bird Island; PG-B, Przewalski’s gazelle on Bird Island; PG-H, Przewalski’s gazelle in Haergai; PG-S, Przewalski’s gazelle in Shengge Township; and TG-S, Tibetan gazelle in Shengge Township.

**Figure 3:**
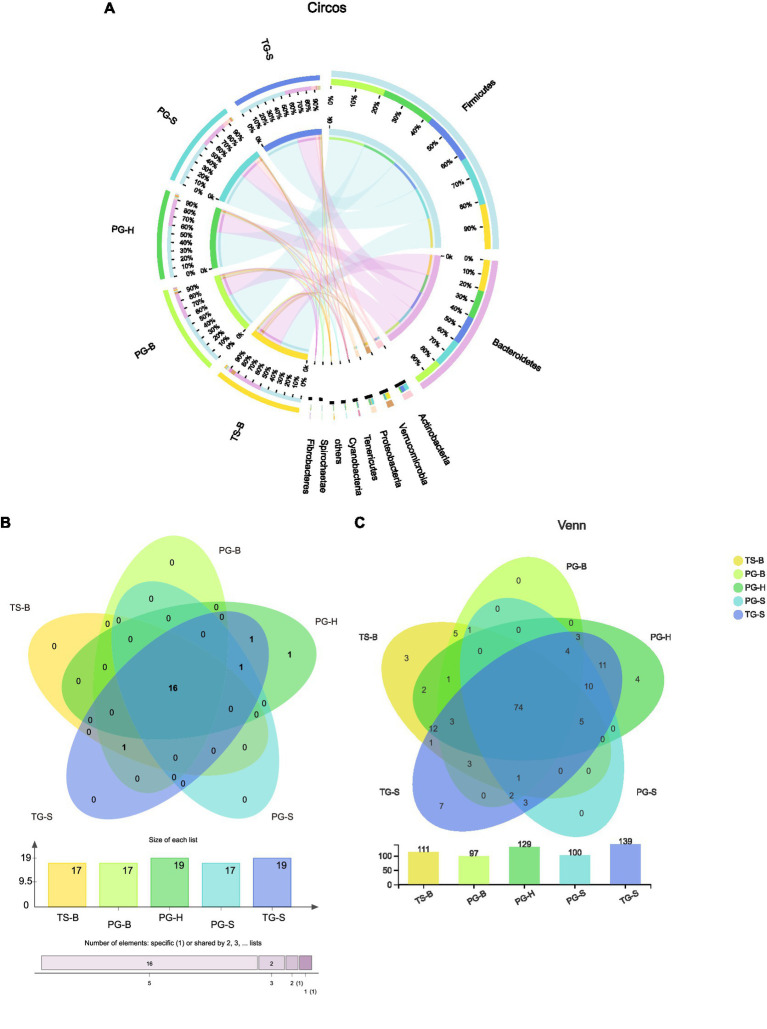
**(A)** Proportion of dominant species in each sample. The outer circle segments of left half indicate different populations, the inner circle of left half indicate bacterial phyla and their abundance in each population. The outer circle segments of right half indicate different phyla and the length of each segment represents the abundance of the phyla. The width of a chord represents the abundance of a phylum in a population. **(B)** Shared and unique phyla of the five populations of the three ruminants. **(C)** Shared and unique families of the five populations of the three ruminants.

We obtained a total of 3,034,642 effective bacterial 16S rRNA gene sequences, ranging from 17,765 to 60,508 sequences in each fecal sample (*n*=50). These sequences were assigned to 2,294 bacterial OTUs at 97% similarity, varying from 957 to 1,451 in each sample. Most of bacterial phylotypes present in the samples were identified according to the rarefaction curves (Appendix S2), numbers of observed OTU richness, alpha diversity indices (Shannon, Simpson, ACE, and Chao1; Appendix S1), and good’s coverage for the fecal bacterial community in each fecal sample. The phylogenetic classification of bacterial sequences affiliated with 20 bacteria phyla, 44 classes, 92 orders, 155 families, and 322 genera. The predominant phyla in all five populations of the three species were Firmicutes (37.27–72.82%) and Bacteroidetes (5.85–44.57%), followed by seven other phyla that represented more than 0.01% of the total sequences in every sample, including Actinobacteria (0.02–41.46%), Verrucomicrobia (0.02–19.29%), Proteobacteria (0.13–10.92%), Tenericutes (0.11–2.50%), Cyanobacteria (0.04–2.62%), Spirochaetae (0.02–7.12%), and Saccharibacteria (0.05–1.67%; Table 2). Fibrobacteres (0.01–12.72%) was higher in the samples of TS-B and PG-B than in other samples (<0.01%), except two TG-S samples. Chloroflexi also varied much among samples, with the highest level of 1.38% found in PG-H (Figures 2A, 3A; Table 2). At family level, Ruminococcaceae (41.14%), Rikenellaceae (13.20%), and Bacteroidaceae (8.60%) were dominant, followed by Lachnospiraceae (6.55%), Christensenellaceae (6.17%), and Prevotellaceae (3.55%; Figure 2B; Table 3).

**Table 2:** Percent of community abundance on phylum level in each population.

	TS-B (mean±SD)	PG-B (mean±SD)	PG-H (mean±SD)	PG-S (mean±SD)	TG-S (mean±SD)
Firmicutes	50.956%±6.681%	61.385%±4.836%	61.203%±5.279%	55.315%±6.626%	57.258%±5.287%
Bacteroidetes	35.237%±4.660%	31.090%±3.871%	31.883%±4.671%	31.313%±2.880%	31.639%±9.535%
Actinobacteria	0.089%±0.035%	0.075%±0.038%	1.988%±1.760%	6.312%±3.285%	6.127%±12.197%
Verrucomicrobia	4.641%±5.246%	2.589%±1.442%	0.714%±0.467%	0.910%±1.262%	0.953%±0.902%
Proteobacteria	1.865%±1.026%	0.889%±0.466%	0.657%±0.458%	3.783%±5.074%	1.217%±1.252%
Tenericutes	1.070%±0.559%	0.694%±0.222%	1.014%±0.689%	0.902%±0.266%	1.079%±0.417%
Cyanobacteria	1.262%±0.739%	1.330%±0.698%	0.525%±0.694%	0.277%±0.386%	0.980%±0.708%
Fibrobacteres	2.873%±3.245%	0.329%±0.430%	–	–	0.004%±0.003%
Spirochaetae	1.074%±0.694%	0.410%±0.401%	1.188%±2.020%	0.477%±0.153%	0.274%±0.183%
Saccharibacteria	0.295%±0.134%	0.838%±0.289%	0.449%±0.467%	0.457%±0.242%	0.277%±0.226%
Chloroflexi	0.008%±0.006%	0.004%±0.000%	0.205%±0.478%	0.009%±0.006%	0.042%±0.063%
Others	0.634%±0.293%	0.369%±0.147%	0.244%±0.202%	0.252%±0.043%	0.135%±0.091%

Phyla with less than 1% abundance were merged into others. Blank means insufficient abundance for statistical purpose. TS-B, Tibetan sheep on Bird Island; PG-B, Przewalski’s gazelle on Bird Island; PG-H, Przewalski’s gazelle in Haergai; PG-S, Przewalski’s gazelle in Shengge Township; and TG-S, Tibetan gazelle in Shengge Township.

**Table 3:** Percent of community abundance on family level in each population.

	TS-B (mean±SD)	PG-B (mean±SD)	PG-H (mean±SD)	PG-S (mean±SD)	TG-S (mean±SD)
Ruminococcaceae	31.130%±5.860%	41.906%±3.749%	44.765%±6.533%	39.687%±4.885%	40.893%±7.413%
Rikeneliaceae	11.896%±2.641%	13.125%±1.920%	12.642%±4.440%	14.348%±1.848%	13.078%±3.975%
Bacteroidaceae	8.147%±1.953%	7.993%±2.240%	10.079%±3.418%	7.654%±1.558%	10.802%±6.070%
Christensenellaceae	7.455%±2.276%	6.238%±2.034%	6.787%±2.876%	5.309%±1.405%	6.616%±2.934%
Lachnospiraceae	7.971%±2.390%	8.878%±3.189%	4.634%±1.577%	5.472%±1.876%	5.386%±1.356%
Prevotellaceae	5.517%±4.339%	3.164%±1.902%	3.393%±2.779%	3.473%±2.919%	1.767%±1.371%
Micrococcaceae	0.017%±0.016%	0.061%±0.023%	1.690%±1.698%	5.985%±3.247%	8.348%±12.054%
Verrucomicrobiaceae	4.457%±5.177%	2.523%±1.435%	0.594%±0.493%	0.854%±1.229%	0.814%±0.793%
Bacteroidales_Incertae_Sedis	3.007%±1.209%	1.807%±0.584%	1.707%±1.233%	1.246%±0.754%	1.081%±1.144%
Bacteroidales_S24-7_group	0.906%±0.517%	1.926%±1.273%	1.320%±0.812%	1.379%±0.305%	2.217%±1.247%
Bacteroidales_BS11_gut_group	1.615%±0.488%	1.710%±0.933%	1.764%±1.938%	0.535%±0.245%	0.666%±0.577%
Acidaminococcaceae	1.177%±0.701%	1.166%±0.847%	1.146%±1.129%	1.297%±0.716%	0.803%±0.560%
norank_o_Mollicutes_RF9	0.978%±0.558%	0.586%±0.232%	0.835%±0.637%	0.818%±0.277%	0.951%±0.359%
norank_o_Gastranaerophilales	1.262%±0.741%	1.320%±0.696%	0.577%±0.699%	0.256%±0.392%	0.926%±0.754%
Fibrobacteraceae	2.874%±3.245%	0.374%±0.441%	–	–	0.047%±0.059%
Porphyromonadaceae	0.469%±0.228%	0.891%±0.365%	0.609%±0.422%	1.114%±0.388%	0.757%±0.593%
Spirochaetaceae	1.078%±0.688%	0.410%±0.403%	1.188%±2.021%	0.476%±0.152%	0.273%±0.185%
Family_XIII	0.602%±0.145%	0.703%±0.080%	0.930%±0.227%	0.629%±0.122%	0.694%±0.333%
Clostridiales_vadinBB60_group	0.485%±0.293%	0.796%±0.531%	0.787%±0.685%	0.412%±0.202%	0.611%±0.603%
Bacteroidales_UCG-001	3.318%±3.228%	0.102%±0.069%	–	–	–
Enterobacteriaceae	0.079%±0.056%	0.033%±0.028%	0.083%±0.074%	3.937%±5.381%	0.267%±0.276%
Desulfovibrionaceae	0.983%±0.387%	0.483%±0.242%	0.272%±0.168%	0.323%±0.213%	0.384%±0.150%
Peptococcaceae	0.491%±0.124%	0.510%±0.123%	0.536%±0.356%	0.321%±0.165%	0.462%±0.250%
Unclassified_c_Bacteroidia	0.531%±0.310%	0.276%±0.356%	0.246%±0.240%	0.022%±0.022%	1.977%±4.503%
Unknown_Class_p_Saccharibacteria	0.295%±0.134%	0.839%±0.282%	0.439%±0.461%	0.457%±0.244%	0.277%±0.226%
Unclassified_o_Bacteroidales	0.161%±0.181%	0.177%±0.196%	0.243%±0.410%	1.537%±1.801%	0.164%±0.175%
Erysipelotrichaceae	0.238%±0.062%	0.344%±0.113%	0.419%±0.172%	0.311%±0.064%	0.480%±0.319%
Peptostreptoceccaceae	0.434%±0.206%	0.071%±0.052%	0.328%±0.281%	0.412%±0.317%	0.359%±0.468%
Rhodospirillaceae	0.301%±0.118%	0.280%±0.204%	0.100%±0.081%	0.083%±0.048%	0.585%±1.229%
Planococcaceae	0.034%±0.021%	0.016%±0.014%	0.415%±0.672%	0.505%±0.639%	0.638%±1.189%
Coriobacteriaceae	0.057%±0.025%	0.029%±0.015%	0.082%±0.092%	0.123%±0.110%	0.323%±0.598%
M2PB4-65_termite_group	0.775%±0.706%	–	–	–	–
Campylobacteraceae	0.588%±0.674%	–	–	–	–
Bacteroidales_RF16_group	0.194%±0.158%	0.010%±0.007%	0.034%±0.027%	–	0.864%±1.040%
norank_o_Opitutae_vadinHA64	0.092%±0.145%	0.026%±0.022%	0.159%±0.288%	0.076%±0.083%	0.330%±0.647%
Nocardiaceae	–	–	0.032%±0.008%	0.030%±0.007%	–
Others	2.165%±0.347%	1.473%±0.239%	1.804%±1.491%	1.579%±0.257%	1.530%±1.682%

Family with less than 1% abundance were merged into others. Blank means insufficient abundance for statistical purpose. TS-B, Tibetan sheep on Bird Island; PG-B, Przewalski’s gazelle on Bird Island; PG-H, Przewalski’s gazelle in Haergai; PG-S, Przewalski’s gazelle in Shengge Township; and TG-S, Tibetan gazelle in Shengge Township.

The shared and unique bacterial taxa between these ruminants were analyzed to find which taxa contributed to the similarities and differences. Among all 20 bacterial phyla, 16 were shared by all the three ruminants from each population (Figures 3B,C). Parcubacteria was found only in Przewalski’s gazelles from Haergai River (PG-H), Nitrospirae was shared by PG-H and Tibetan gazelles from Shengge (TG-S), Synergistetes was shared by Przewalski’s gazelles from Haergai and Shengge (PG-H and PG-S) and Tibetan gazelles from Shengge (TG-S), but these three phyla had very low abundance. Fibrobacteres was abundant in Przewalski’s gazelles and Tibetan sheep from Bird Island (PG-B, TS-B) but was also found in several samples from Tibetan gazelles from Shengge (TG-S; Table 2).

### Comparison Among Przewalski’s Gazelle Populations

**Figure 4:**
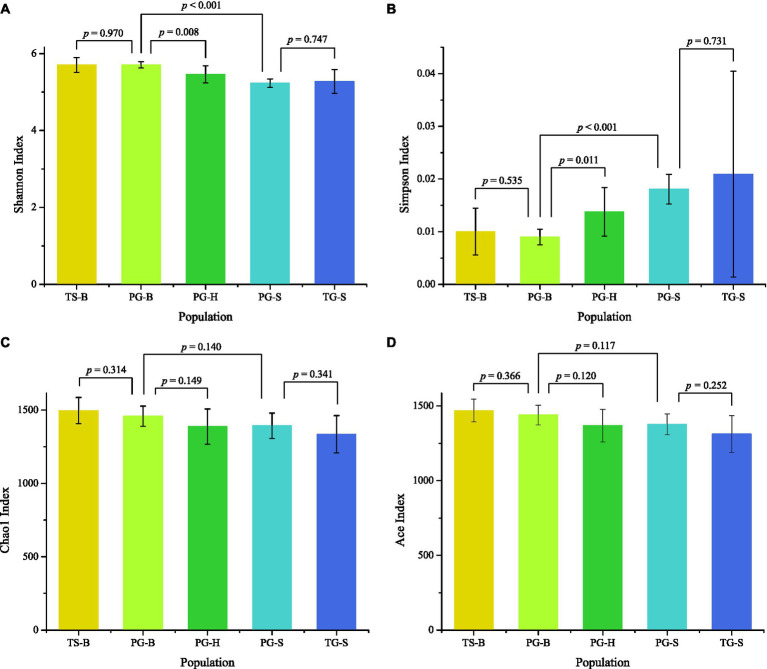
Comparison of alpha diversity of the gut bacterial community among the five populations. TS-B (yellow), Tibetan sheep on Bird Island; PG-B (lime), Przewalski’s gazelle on Bird Island; PG-H (green), Przewalski’s gazelle in Haergai; PG-S (mint), Przewalski’s gazelle in Shengge Township; and TG-S (blue), Tibetan gazelle in Shengge Township. Diversity **(A,B)** was significantly higher in managed PG-B than in wild PG-H and PG-S, but richness **(C,D)** was not different. No differences were found between sympatric PG-B and TS-B, and between sympatric PG-S and TG-S.

**Figure 5:**
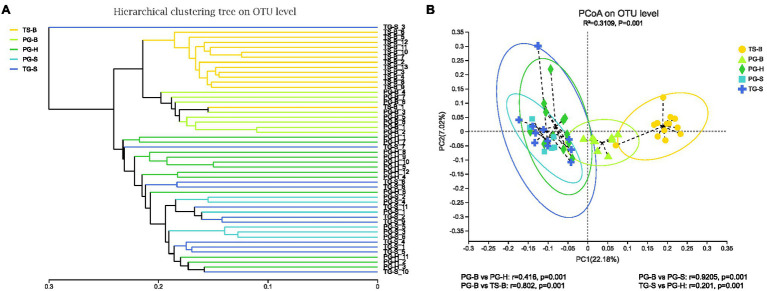
Comparison of the gut bacterial community of the three species from five populations. **(A)** Clustering analysis of the evolution of the gut bacterial community of the isolated Przewalski’s gazelle in Haergai (PG-H) and sympatric Przewalski’s gazelle (PG-S) and Tibetan gazelle in Shengge (TG-S). The managed population of Przewalski’s gazelles (PG-B) was sympatric and mixed with Tibetan sheep on Bird Island (TS-B). Gut bacterial trees were generated using the unweighted pair group method with arithmetic mean (UPGMA) algorithm based on the unweighted-unifrac distances generated by mothur. The managed PG-B and sympatric Tibetan sheep clustered on the same branch, while three wild gazelle populations clustered together. **(B)** Principal coordinate analysis (PCoA) of microbial communities. Distances between symbols on the ordination plot reflect relative dissimilarities in community structures. Paired differences of analysis of similarities (ANOSIM) were shown below. Gut bacterial community of managed Przewalski’s gazelle (PG-B) was significantly different with that of their wild relatives.

**Figure 6:**
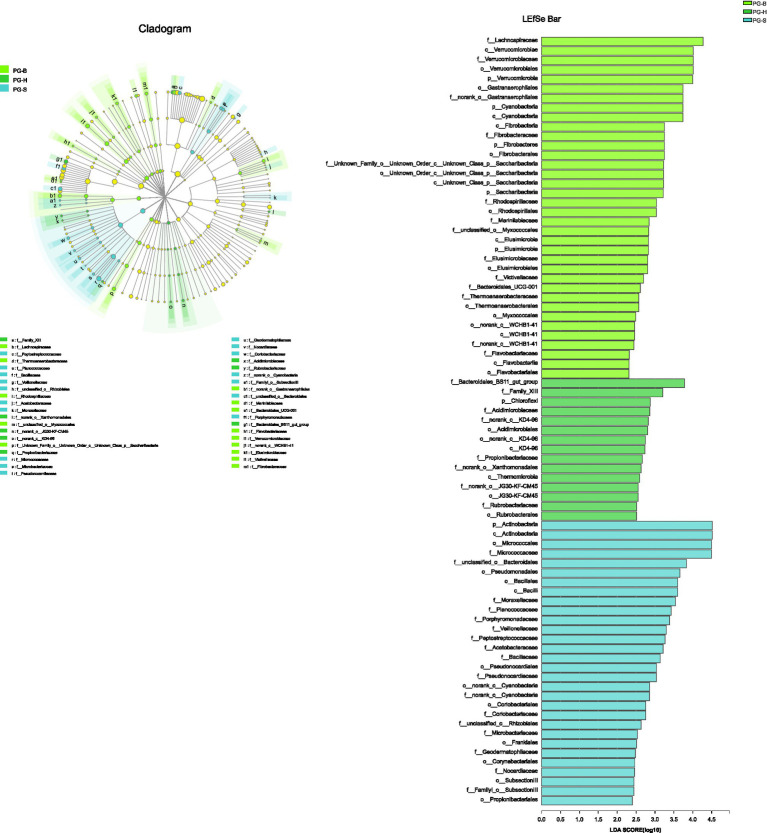
The phyla to families that contribute to the differences between Przewalski’s gazelle populations identified by linear discriminant analysis coupled with effect size (LEfSe). PG-B (lime) is short for Przewalski’s gazelle from Bird Island, PG-H (green) is short for Przewalski’s gazelle from Haergai River and PG-S (mint) is short for Przewalski’s gazelle from Shengge Township.

The richness (Chao1, ACE) of the fecal bacterial community did not vary between the wild and managed populations of Przewalski’s gazelles, but the diversity (Shannon, Simpson) of the fecal bacterial community was higher in the managed than the wild populations (PG-B vs. PG-H: Shannon *p*=0.008, Simpson *p*=0.011; PG-B vs. PG-S: Shannon *p*<0.001, Simpson *p*<0.001; Figure 4; Table 4). Przewalski’s gazelles at Haergai (PG-H) and Shengge (PG-S) clustered together with Shengge Tibetan Gazelles (TG-S), but were significantly distinct from Przewalski’s gazelles at Bird Island (PG-B) using a hierarchical clustering analysis (Figure 5A). Similarly, the wild populations of Przewalski’s gazelles (PG-H and PG-S) were distinct from the managed PG-B population in accordance with the bacterial community structure on PC1 of the PCoA plot, which accounted for 22.18% of the total variation, with a little overlap. PG-H and PG-S could not be separated (Figure 5B). The similarity (ANOSIM) of the five populations was 0.6141 (*p*=0.001). While PG-B was significantly different from those from PG-H (*r*=0.416, *p*=0.001) and PG-S (*r*=0.9205, *p*=0.001), the two wild Przewalski’s gazelle populations were not different (PG-S vs. PG-H: *r*=0.131, *p*=0.136). The LEfSe analysis identified the taxa which contributed to the differences of the three populations (Figure 6; Appendix S3). Specifically, Chloroflexi was more abundant in the fecal samples from PG-H, Actinobacteria more abundant in PG-S, while Verrucomicrobia, Cyanobacteria, Fibrobacteres, Saccharibacteria, and Elusimicrobia were more abundant in PG-B, the managed population. At family level, we found 39 families were differentially represented in the three populations using LEfSe (Figure 6).

**Table 4:** One-way ANOVA comparison (significant value) of diversity indices of fecal bacterial community in two wild Przewalski’s gazelle populations (PG-H and PG-S), one managed Przewalski’s gazelle population (PG-B), one Tibetan gazelle population (TG-S), and one Tibetan sheep population (TS-B).

Index	Population	PG-B	PG-H	PG-S	TS-B
Shannon	PG-H	0.008395^*^			
Simpson		0.01126^*^			
ACE		0.1199			
Chao1		0.1487			
Shannon	PG-S	<0.0001^*^	0.03365^*^		**<0.0001**
Simpson		<0.0001^*^	0.05448		**0.00082**
ACE		0.1169	0.8598		**0.02288**
Chao1		0.1398	0.9204		**0.0293**
Shannon	TS-B	0.9702	**0.007505**		
Simpson		0.5351	**0.04881**		
ACE		0.3658	**0.01298**		
Chao1		0.3135	**0.01602**		
Shannon	TG-S	0.001382^*^	0.115	0.7466	**0.000422**
Simpson		0.1053	0.2319	0.7313	**0.06265**
ACE		0.01691^*^	0.2536	0.2515	**0.000907**
Chao1		0.02435^*^	0.3261	0.3413	**0.001442**

* These two populations are significantly different.

Bold values indicate the comparison has no ecological meaning.

### Comparison Between Przewalski’s Gazelle and Sympatric Ruminants

We found no significant differences in alpha-diversity of the fecal bacterial communities between sympatric Prezewalski’s gazelles (PG-B) and Tibetan sheep (TS-B), or between Prezewalski’s gazelles and sympatric Tibetan gazelles from Shengge (Table 4). Notably, both Prezewalski’s gazelles and Tibetan sheep from Bird Island clustered on the same branch in the dendrogram (Figure 5A), indicating that the microbiota of PG-B was more similar to that of the sympatric TS-B than to their allopatric conspecifics. However, PG-B and TS-B were still distinguishable from each other in the PCoA plot, as well as by ANOSIM (PG-B vs. TS-B: *r*=0.802, *p*=0.001). Furthermore, microbiota was more similar for the sympatric wild *Procapra* populations gazelles (PG-S vs. TG-S: *r*=0.0268, *p*=0.372) than for the allopatric gazelles (TG-S vs. PG-H: *r*=0.201, *p*=0.001).

**Figure 7:**
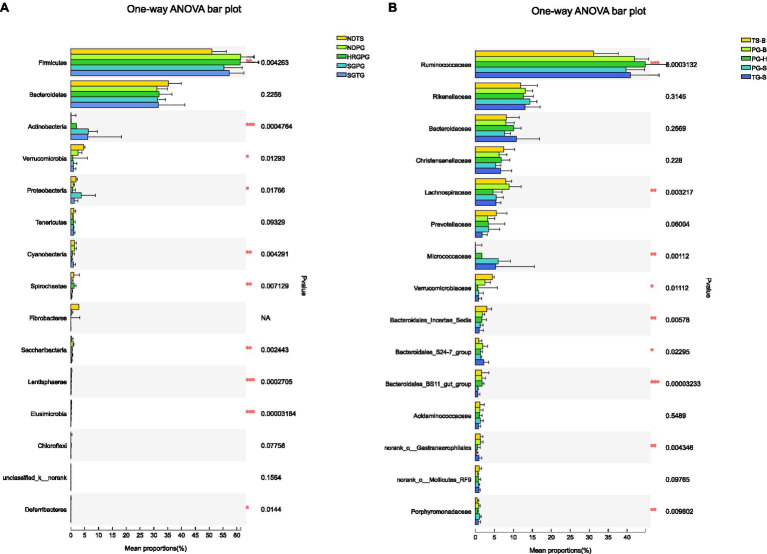
**(A)** Phyla differences among the Przewalski’s gazelle from Haergai (PG-H), Bird Island (PG-B), Shengge Township (PG-S), Tibetan gazelle from Shengge Township (TG-S), and Tibetan sheep from Bird Island (TS-B). **(B)** Family differences among the Przewalski’s gazelle from Haergai (PG-H), Bird Island (PG-B), Shengge Township (PG-S), Tibetan gazelle from Shengge Township (TG-S), and Tibetan sheep from Bird Island (TS-B).

**Figure 8:**
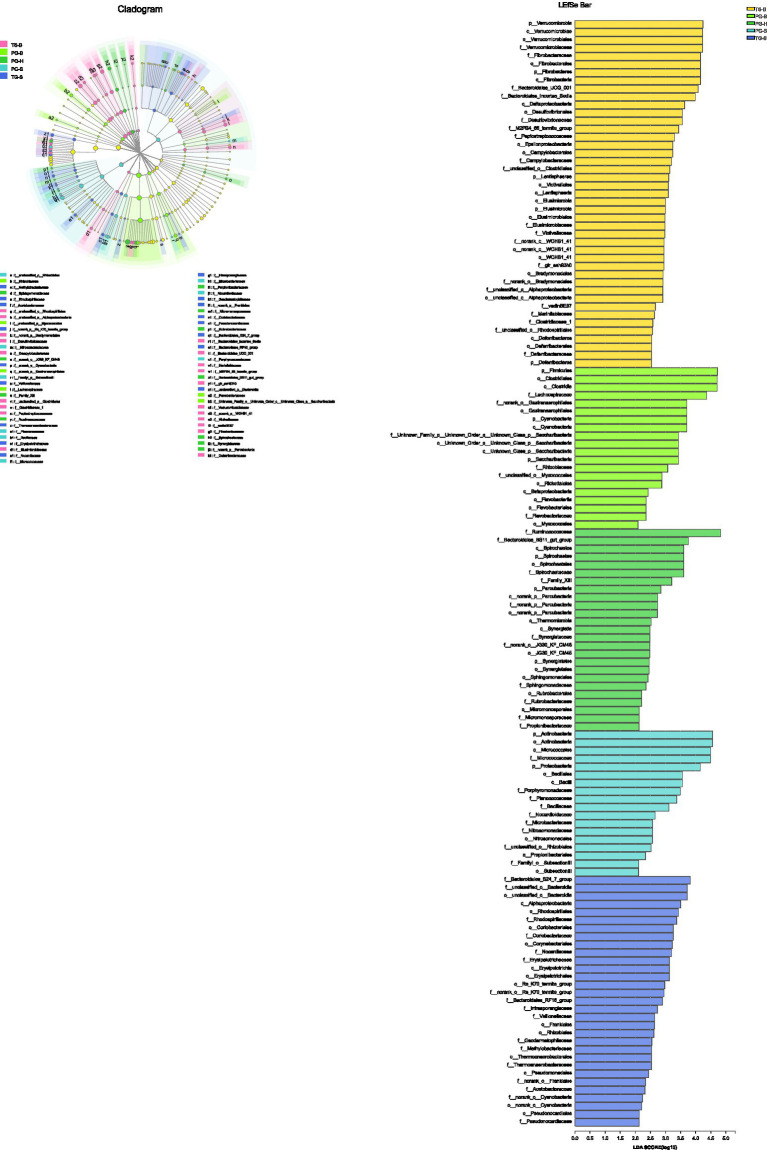
The phyla to families that contribute to the differences among populations identified by LEfSe.

One-way ANOVA of multiple groups showed that 14 out of the 20 phyla were significantly different between populations (Figure 7A). At the family level, Ruminococcaceae, Lachnospiraceae, Micrococcaceae, Bacteroidales, and other five families were different between populations (Figure 7B). Combining the result of LEfSe, we found that the relative abundance of 11 phyla contributed to the difference of each population, except for TG-S (Figure 8). When species and environment were combined into the ANOVA, there was no significant effect of either on alpha-diversity indices, except for Shannon diversity, where environment (*F*_2,4_=8.566, *p*=0.001), but not species (*F*_1,4_=0.161, *p*=0.690) predicted diversity.

Combining the results from intra-species comparison of Przewalski’s gazelles, we found that the fecal bacterial community of PG-B shifted to resemble that of sympatric Tibetan sheep (TS-B): several bacterial genera such as *Desulfovibrio*, *Spirochete*, and *Clostridium* did not exist in their wild relatives (PG-S or PG-H).

## Discussion

Our results reveal that both heritable factors and ecological factors shaped the gastrointestinal bacterial community of Przewalski’s gazelle and the sympatric Tibetan gazelle and Tibetan sheep. The microbiota of managed Przewalski’s gazelle that co-occur with Tibetan sheep had higher diversity and included taxa that were not present in wild populations of either Przewalski’s or Tibetan gazelles. This variation across populations could be the result of either shared environments such as diet or from direct fecal exposure between species (Moeller et al., 2013; Hale et al., 2018), which could be distinguished by sampling additional populations or using natural experiments.

### Bacterial Diversity in Wild and Managed Przewalski’s Gazelles

Ruminants have enlarged gastrointestinal tracts that provide an anaerobic environment to harbor highly complex symbiotic gastrointestinal microbes (Morgavi et al., 2013). These sophisticated fermentation systems allow ruminants to adapt to a large variety of habitats and diets (Morgavi et al., 2013). As a typical ruminant, Przewalski’s gazelle’s fecal bacterial communities are dominated by Firmicutes and Bacteroidetes, which contain many fibrolytic species. For example, Ruminococcaceae and Lachnospiraceae are two of the most abundant families from the phylum Firmicutes and can ferment diverse plant structural carbohydrates to SCFAs and alcohols. Prevotellaceae from phylum Bacteroidetes can breakdown proteins and carbohydrates into propionate or succinate and acetate, which may contribute to the abundance of SCFAs in high-altitude ruminants (Zhang et al., 2016). In addition, Proteobacteria and Verrucomicrobia also contain species that have genes encoding putative glycosyl-hydrolases for metabolize cellulose (Borbón-García et al., 2017).

Although, the gut bacterial richness was similar for the wild and managed populations of Przewalski’s gazelle, the bacterial diversity was different. The fecal samples from the managed PG-B contained more abundant Verrucomicrobia, Cyanobacteria, Fibrobacteres, Saccharibacteria, and Elusimicrobia than those from the wild PG-S and PG-H. Verrucomicrobia is believed to contribute to intestinal health and glucose homeostasis, and its abundance is negatively correlated with body mass in human (Solar et al., 2019). Fibrobacteres may have a role in cellulose degradation (Rahman et al., 2016), whereas Saccharibacteria and Elusimicrobia are two widespread, but poorly known phyla. Cyanobacteria, originally referred to as the “blue-green algae,” are the only recognized prokaryotes that exhibit photosynthesis with the generation of oxygen and the only oxygenic microorganisms that can also fix nitrogen (Percival and Williams, 2013). Non-photosynthetic Cyanobacteria are present in the ruminant gut and have recently been designated to a new candidate class named Melainabacteria (Soo et al., 2014; Neves et al., 2017). We are not sure where the Cyanobacteria in the managed PG-B originated, but the presence of abundant Cyanobacteria in these animals warrants conservation concern and needs to be further investigated.

At family level, we found that Lachnospiraceae and Gastranaerophilales were rich in PG-B and TS-B, while Micrococcaceae were rich in PG-S and TG-S. Lachnospiraceae is able to utilize diet-derived polysaccharides, including starch, inulin, and arabinoxylan, with substantial variability among species and strains (Vacca et al., 2020). High abundance of Lachnospiraceae is positively correlated with glucose and/or lipid metabolism, indicating metabolic disturbance (Vacca et al., 2020). The Gastranaerophilales are found in human and other animal guts, although their exact role is unknown (Monchamp et al., 2019). Meanwhile, Gastranaerophilales and Lachnospiraceae are positively correlated with non-alcoholic fatty liver disease in human, and may cause lipid accumulation, liver injury, hyperglycemia, and inflammation (Chi et al., 2019; Vacca et al., 2020). The enriched Lachnospiraceae and Gastranaerophilales found in PG-B and its sympatric TS-B may mean that they use different routes to generate energy from fermentable substrates, probably due to their different diets under managed conditions. Like many other representatives of the Actinobacteria, Micrococcaceae has the ability to utilize a wide range of unusual substrates (Dastager et al., 2014). Members of the Micrococcaceae family have been isolated from various habitats, including activated sludge, medieval wall painting, meat, human and other mammal skin, marine sediment, freshwater, desert soil, cyanobacterial mat, plants, seafood, saline soil, and oral cavity from which the original cultures were isolated (Dastager et al., 2014). We need further research to explain why Micrococcaceae was elevated in PG-S and TG-S.

### Do Microbes Show Convergence in Sympatric Relatives or Are They Species-Specific?

The comparison of the fecal bacterial community among the five populations of three ruminants indicated that both heritable and ecological factors impact the gut bacterial community. Phylogeny appears to play an important role as the clustering analysis and PCoA, together with alpha-diversity comparison, showed that the fecal bacterial communities of PG-H and TG-S were generally similar, even though, they are different species from different locations. The three wild *Procapra* gazelle populations are phylogenetically close relatives, and have similar body weight and behavior (Li et al., 2008, 2010), which might lead to similar gastrointestinal structure and bacterial communities among them. The managed Przewalski’s gazelles (PG-B), on the other hand, were significantly different in alpha-diversity compared to their relatives from the two wild populations (PG-S and PG-H), but there was no difference with the sympatric Tibetan sheep (TS-B). However, the result of beta-diversity comparison and ANOSIM indicated that, although partly similar, the fecal bacterial community of PG-B could be separated from TS-B, suggesting heritable, or phylogenetic, component to the fecal bacterial community.

A key finding was that the fecal bacterial communities of Przewalski’s gazelle and Tibetan sheep converge where they are sympatric. Two explanations for this similarity are dietary overlap and contact with excreted bacteria. Dietary overlap is a likely explanation for the similarity. Previous studies of different types of mammals have revealed that their fecal microbiota and function cluster according to diet (herbivores, omnivores, and carnivores) rather than host phylogeny (Ley et al., 2008; Muegge et al., 2011). The microbiome composition of Rocky Mountain elk (*Cervus canadensis nelsoni*) that was supplied with alfalfa pellets changed compared to that in natural feed population (Couch et al., 2021b). In captive primates, gut microbiota cluster weakly by species, but strongly by diet, so that the gut microbiota is more similar when monkeys in the same genus are fed with the same diet, even if they are different species (Hale et al., 2018; Huan et al., 2020). A comparative study of sympatric and allopatric populations of the genera *Pan* and *Gorilla* has found that, despite the strong influence of host phylogenetic history on their gut microbiota, the gut microbiota of *Pan* and *Gorilla* converge where the two genera are sympatric, indicating that sharing environment and diet overlap play important roles in determining the composition of gut microbiota in great ape (Moeller et al., 2013). The microscopic fecal analysis of the three ruminants reveals that Przewalski’s gazelles and Tibetan gazelles forage similar diets even if living separately, while the diets of Tibetan sheep and the two gazelle species are highly overlapped (Li et al., 2008). Przewalski’s gazelle and Tibetan sheep living in sympatry might therefore cultivate similar gut environments due to their shared diets, potentially favoring specific bacterial constituents. Another possible source of the convergence might be that bacterial phylotypes could be transferred between hosts of different species. High similarity in microbiome communities between sympatric terrestrial species with different diets relative to closely related species with similar diets has been documented in Malagasy mammals (Perofsky et al., 2019). We did not record direct contact among individuals of different species in the field, but their shared environment might lead to indirect transfer, e.g., the contamination of bacteria from feces of another species in water, soil, or vegetation. A better understanding of the potential for pathogen transmission through fecal contact is important for managing mixed grazing landscapes, for both wildlife and livestock health.

Such taxonomic convergence in bacterial composition of Przewalski’s gazelle and Tibetan sheep suggests the possibility of transmission and sharing of pathogens between domestic and wild animals, which has important implication for the health of wildlife in captivity. *Desulfovibrio* was found only in managed Przewalski’s gazelle and Tibetan sheep. This genus was first isolated from sheep rumen as the predominant sulfate reducer that can convert sulfate and other oxyanions of sulfur in the gut into hydrogen sulfide, a cytotoxic and genotoxic gas that has been linked to inflammatory bowel disease and colorectal cancer in humans (Howard and Hungate, 1976; Amato et al., 2016). *Leptospira*, a member of another potential pathogen *Spirochete*, can cause leptospirosis that principally infects domestic and wild mammals and is a secondary infection of humans (Gupta et al., 2013). Many species in genus *Clostridium* were thought to be butyric acid producer, and can stabilize ruminal pH by consume lactate (Miguel et al., 2019; Cai et al., 2021). However, *Clostridium* also includes several significant pathogens which can cause Clostridial abomasitis and enteritis in all species of domestic ruminants (Simpson et al., 2018), and can cause the causative agents of botulism and tetanus in human (Cruz-Morales et al., 2019). Since microbes play important roles in many biological processes that affect host health and function, microbiome divergence from that of wild conspecifics may lead to dysbiosis, which in turn reduces functional capacity of the host gut microbiome and represents potentially severe health implications for threatened species like Przewalski’s gazelle (West et al., 2019). The similarity of the gut bacterial community of sympatric Przewalski’s gazelles and Tibetan gazelles could be because they are phylogenetically close relatives that have similar gut morphology, immune system, and diet, which in turn lead to few differences in their gut bacterial community.

In conclusion, our study has characterized the fecal bacterial community of two wild populations and one managed population of Przewalski’s gazelle, along with two sympatric ruminants, the Tibetan gazelle and Tibetan sheep. Our results find that the bacterial communities of congeneric populations were more similar than those of heterogeneric populations, and the bacterial communities of different host species were distinguishable from one another. Furthermore, the influence of ecological factors on bacterial community was significant. Our study also provides useful implications for conservation management of captive wildlife populations, i.e., it is important to monitor the microbiome function and stability in captive animals in order to maintain their digestion efficiency and to increase their resistance to pathogens and enteric disease. Meanwhile, wildlife may be exposed to domestic animals when kept in conservation areas or breeding centers, in which transmission of pathogens and diseases between livestock and wildlife is possible. Such transmission could result in serious consequences for captive individuals, or lead to infection in wild populations when such captive individuals are reintroduced to their natural habitat. Considering this, the current study serves as a first step toward the successful application of next-generation sequencing techniques in the context of conservation of the endangered Przewalski’s gazelle. Since our study was conducted in wild populations of the endangered species, the sample size was relatively small. Limited by the condition of field station, we did not use cryopreservation at −80°C or other strict storage method like liquid nitrogen. Further studies may focus on quantification of susceptive bacterial taxa and exploring the specific factors that influence Przewalski’s gazelle’s gut health. Moreover, studies on parasites and gut microbiome, and their impact on gazelle’s survival, will have significantly importance for this precious and mysterious species.

## Data Availability Statement

The datasets presented in this study can be found in online repositories. The names of the repository/repositories and accession number(s) can be found at: https://www.ncbi.nlm.nih.gov/, PRJNA684634.

## Ethics Statement

The animal study was reviewed and approved by Law of the People’s Republic of China on the Protection of Wildlife.

## Author Contributions

RL: conceptualization, methodology, formal analysis, investigation, writing – original draft, and visualization. JS: conceptualization, writing – review and editing, supervision, project administration, and funding acquisition. SS: writing – review and editing. DG and DL: resources. All authors contributed to the article and approved the submitted version.

## Funding

This work was funded by the National Natural Science Foundation of China (Project No.: 31572281) and the Interdisciplinary Research Funds of Beijing Normal University. SS is supported by a Royal Society URF (UF160725).

## Conflict of Interest

The authors declare that the research was conducted in the absence of any commercial or financial relationships that could be construed as a potential conflict of interest.

## Publisher’s Note

All claims expressed in this article are solely those of the authors and do not necessarily represent those of their affiliated organizations, or those of the publisher, the editors and the reviewers. Any product that may be evaluated in this article, or claim that may be made by its manufacturer, is not guaranteed or endorsed by the publisher.

## Footnotes

^1^ www.i-sanger.com

^2^ http://drive5.com/uparse/

^3^ http://sourceforge.net/projects/rdp-classifier/

^4^ https://mothur.org/wiki/summary.single/

^5^ https://CRAN.R-project.org/package=VennDiagram
