# Supplementary material for: Fecal Bacterial Community of Allopatric Przewalski’s Gazelles and Their Sympatric Relatives
Source: Front Microbiol. 2021 Sep 24;12:737042. doi: 10.3389/fmicb.2021.737042 (PMC8499116; doi:10.3389/fmicb.2021.737042)
Supplement: Supplementary file 1 [file Data_Sheet_1.doc]

Supplementary Information for

Fecal bacterial community of allopatric Przewalski’s gazelles and their sympatric relatives

**Appendix S1.** Diversity indices of all fecal samples from two wild Przewalski’s gazelle populations (PG-H, PG-S), one captive Przewalski’s gazelle population (PG-B), one Tibetan gazelle population (TG-S), and one Tibetan sheep population (TS-B).

| Sample\Estimators | sobs | shannon | simpson | ace | chao | coverage |
| --- | --- | --- | --- | --- | --- | --- |
| PG-H_1 | 1023 | 5.028798 | 0.023527 | 1252.355 | 1233.734 | 0.991668 |
| PG-H_10 | 1153 | 5.415523 | 0.015069 | 1433.545 | 1511.693 | 0.984445 |
| PG-H_11 | 1089 | 5.464918 | 0.011294 | 1270.321 | 1275.388 | 0.993446 |
| PG-H_12 | 1142 | 5.347377 | 0.014982 | 1329.126 | 1333.265 | 0.991592 |
| PG-H_2 | 1323 | 5.724319 | 0.008874 | 1495.464 | 1498.877 | 0.994097 |
| PG-H_3 | 1289 | 5.443481 | 0.012829 | 1445.588 | 1448.378 | 0.996463 |
| PG-H_4 | 1322 | 5.571304 | 0.012592 | 1480.538 | 1489.739 | 0.994387 |
| PG-H_5 | 1272 | 5.718617 | 0.00943 | 1484.392 | 1535.387 | 0.990086 |
| PG-H_6 | 1126 | 5.429622 | 0.013472 | 1327.247 | 1385.048 | 0.990277 |
| PG-H_7 | 957 | 5.108843 | 0.021722 | 1195.455 | 1222.008 | 0.985139 |
| PG-H_8 | 1103 | 5.719537 | 0.009419 | 1248.541 | 1241.534 | 0.990741 |
| PG-H_9 | 1271 | 5.56645 | 0.012207 | 1458.187 | 1470.238 | 0.991831 |
| PG-B_1 | 1323 | 5.813276 | 0.008361 | 1525.056 | 1533.438 | 0.992136 |
| PG-B_2 | 1302 | 5.796443 | 0.008709 | 1470.99 | 1470.681 | 0.993071 |
| PG-B_3 | 1183 | 5.731191 | 0.008473 | 1407.19 | 1413.35 | 0.988608 |
| PG-B_4 | 1143 | 5.587148 | 0.010308 | 1335.558 | 1334.849 | 0.991483 |
| PG-B_5 | 1264 | 5.665592 | 0.009455 | 1506.689 | 1539.07 | 0.992471 |
| PG-B_6 | 1167 | 5.700438 | 0.007814 | 1369.686 | 1410.344 | 0.990945 |
| PG-B_7 | 1213 | 5.754057 | 0.007051 | 1458.946 | 1490.245 | 0.990351 |
| PG-B_8 | 1217 | 5.616245 | 0.011722 | 1436.917 | 1473.419 | 0.989686 |
| TS-B_1 | 1170 | 5.598873 | 0.009191 | 1366.857 | 1390.075 | 0.991214 |
| TS-B_10 | 1323 | 5.783405 | 0.009774 | 1471.878 | 1489.955 | 0.993211 |
| TS-B_11 | 1282 | 5.542917 | 0.016627 | 1546.198 | 1614.604 | 0.989804 |
| TS-B_12 | 1214 | 5.878175 | 0.00639 | 1456.103 | 1463.189 | 0.987462 |
| TS-B_13 | 1228 | 5.441114 | 0.015901 | 1402.115 | 1431.923 | 0.992242 |
| TS-B_2 | 1372 | 5.741653 | 0.008361 | 1529.366 | 1545.313 | 0.994441 |
| TS-B_3 | 1451 | 5.816993 | 0.006941 | 1616.029 | 1652.194 | 0.995945 |
| TS-B_4 | 1358 | 5.905656 | 0.005795 | 1549.471 | 1602.221 | 0.993286 |
| TS-B_5 | 1183 | 5.844742 | 0.007011 | 1361.586 | 1353.524 | 0.98993 |
| TS-B_6 | 1164 | 5.759514 | 0.007119 | 1410.683 | 1423.586 | 0.986208 |
| TS-B_7 | 1306 | 5.977847 | 0.005211 | 1445.917 | 1479.309 | 0.993095 |
| TS-B_8 | 1276 | 5.483155 | 0.014848 | 1487.202 | 1519.388 | 0.99296 |
| TS-B_9 | 1267 | 5.395208 | 0.017078 | 1452.283 | 1487.745 | 0.994084 |
| PG-S_1 | 1162 | 5.439779 | 0.013685 | 1378.865 | 1409.356 | 0.988621 |
| PG-S_2 | 1129 | 5.141272 | 0.021841 | 1313.646 | 1333.187 | 0.991506 |
| PG-S_3 | 1174 | 5.194193 | 0.016391 | 1337.25 | 1337.5 | 0.995792 |
| PG-S_4 | 1327 | 5.271049 | 0.019414 | 1483.78 | 1531.147 | 0.996095 |
| PG-S_5 | 1229 | 5.16538 | 0.01909 | 1437.464 | 1445.115 | 0.993834 |
| PG-S_6 | 1111 | 5.193112 | 0.018018 | 1312.517 | 1300.062 | 0.991254 |
| TG-S_1 | 1145 | 5.458012 | 0.011834 | 1306.226 | 1350.208 | 0.9933 |
| TG-S_10 | 1302 | 5.525505 | 0.010716 | 1475.238 | 1488.773 | 0.996113 |
| TG-S_11 | 1204 | 5.370275 | 0.015791 | 1371.297 | 1380.289 | 0.99319 |
| TG-S_2 | 1203 | 5.368959 | 0.015694 | 1372.761 | 1405.583 | 0.99564 |
| TG-S_3 | 972 | 4.454403 | 0.078709 | 1047.837 | 1062.093 | 0.995366 |
| TG-S_4 | 1211 | 5.317153 | 0.014459 | 1350.899 | 1366.628 | 0.995176 |
| TG-S_5 | 1225 | 5.470869 | 0.013955 | 1372.247 | 1432.093 | 0.995297 |
| TG-S_6 | 1057 | 5.054434 | 0.02513 | 1211.013 | 1229.55 | 0.99465 |
| TG-S_7 | 994 | 5.168007 | 0.016631 | 1171.364 | 1187.319 | 0.993303 |
| TG-S_8 | 1101 | 5.338545 | 0.014044 | 1319.001 | 1337.257 | 0.991589 |
| TG-S_9 | 1276 | 5.527461 | 0.013148 | 1428.297 | 1448.351 | 0.995912 |


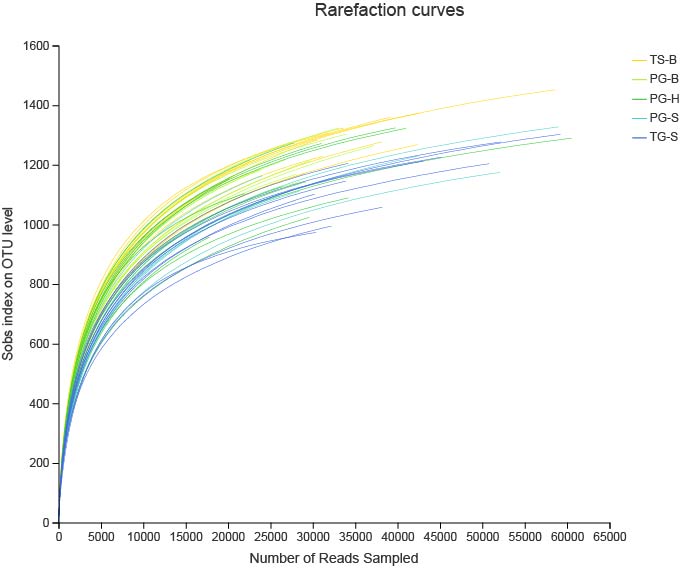
**Appendix S2.** Rarefaction curves of each sample from two wild Przewalski’s gazelle populations (PG-H, PG-S), one managed Przewalski’s gazelle population (PG-B), one Tibetan gazelle population (TG-S), and one Tibetan sheep population (TS-B).

**Appendix S3.** Non-parametric factorial Kruskal-Wallis test in LEfSe of fecal bacterial community in two wild Przewalski’s gazelle populations (PG-H, PG-S), one captive Przewalski’s gazelle population (PG-B), one Tibetan gazelle population (TG-S), and one Tibetan sheep population (TS-B). Only significant species were shown.

| Species name | group | Mean | LDA value | P value |
| --- | --- | --- | --- | --- |
| p__Firmicutes.c__Bacilli | SGPG | 3.889743179 | 3.60170779 | 0.000188 |
| p__Saccharibacteria.c__Unknown_Class_p__Saccharibacteria.o__Unknown_Order_c__Unknown_Class_p__Saccharibacteria.f__Unknown_Family_o__Unknown_Order_c__Unknown_Class_p__Saccharibacteria | NDPG | 3.922668461 | 3.227742665 | 0.024729 |
| p__Actinobacteria.c__Actinobacteria.o__Propionibacteriales | SGPG | 2.555701503 | 2.397454172 | 0.010498 |
| p__Proteobacteria.c__Alphaproteobacteria.o__Rhodospirillales.f__Rhodospirillaceae | NDPG | 3.447541915 | 3.038134704 | 0.007881 |
| p__Actinobacteria.c__Actinobacteria.o__Micrococcales | SGPG | 4.781003107 | 4.498201876 | <0.0001 |
| p__Cyanobacteria.c__Cyanobacteria.o__norank_c__Cyanobacteria | SGPG | 1.879350553 | 2.853611997 | 0.001478 |
| p__Firmicutes.c__Bacilli.o__Bacillales.f__Planococcaceae | SGPG | 3.703022092 | 3.425289514 | 0.00079 |
| p__Firmicutes.c__Clostridia.o__Clostridiales.f__Family_XIII | HRGPG | 3.968378927 | 3.203593709 | 0.009039 |
| p__Bacteroidetes.c__Bacteroidia.o__Bacteroidales.f__unclassified_o__Bacteroidales | SGPG | 4.186603417 | 3.832241287 | 0.023889 |
| p__Actinobacteria.c__Actinobacteria.o__Pseudonocardiales | SGPG | 1.378896072 | 3.038179622 | 0.029403 |
| p__Chloroflexi.c__KD4-96 | HRGPG | 2.236715892 | 2.733438693 | 0.032644 |
| p__Firmicutes.c__Clostridia.o__Clostridiales.f__Lachnospiraceae | NDPG | 4.948341311 | 4.272011843 | 0.009797 |
| p__Proteobacteria.c__Deltaproteobacteria.o__Myxococcales | NDPG | 2.216782217 | 2.488918404 | 0.010035 |
| p__Verrucomicrobia.c__Verrucomicrobiae | NDPG | 4.40195442 | 4.009110372 | 0.002613 |
| p__Saccharibacteria | NDPG | 3.922668461 | 3.218584858 | 0.025937 |
| p__Verrucomicrobia | NDPG | 4.413097883 | 3.996972047 | 0.003117 |
| p__Proteobacteria.c__Gammaproteobacteria.o__Pseudomonadales | SGPG | 1.158822336 | 3.659196438 | 0.004469 |
| p__Actinobacteria.c__Actinobacteria.o__Frankiales.f__Geodermatophilaceae | SGPG | 2.575281928 | 2.476727497 | 0.000511 |
| p__Firmicutes.c__Bacilli.o__Bacillales | SGPG | 3.886720053 | 3.602086907 | 0.000316 |
| p__Chloroflexi.c__Thermomicrobia.o__JG30-KF-CM45.f__norank_o__JG30-KF-CM45 | HRGPG | 2.735319206 | 2.554683537 | 0.029232 |
| p__Firmicutes.c__Clostridia.o__Clostridiales.f__Peptostreptococcaceae | SGPG | 3.614489188 | 3.265517967 | 0.000542 |
| p__Actinobacteria | SGPG | 4.800212952 | 4.513129653 | <0.0001 |
| p__Chloroflexi.c__Thermomicrobia | HRGPG | 2.784094205 | 2.595289625 | 0.029232 |
| p__Verrucomicrobia.c__WCHB1-41.o__norank_c__WCHB1-41.f__norank_c__WCHB1-41 | NDPG | 2.67924679 | 2.441743054 | 0.00744 |
| p__Bacteroidetes.c__Flavobacteriia.o__Flavobacteriales | NDPG | 2.315423525 | 2.309362343 | 0.012397 |
| p__Saccharibacteria.c__Unknown_Class_p__Saccharibacteria.o__Unknown_Order_c__Unknown_Class_p__Saccharibacteria | NDPG | 3.922668461 | 3.227742665 | 0.024729 |
| p__Chloroflexi.c__Thermomicrobia.o__JG30-KF-CM45 | HRGPG | 2.735319206 | 2.554010148 | 0.029232 |
| p__Fibrobacteres.c__Fibrobacteria.o__Fibrobacterales.f__Fibrobacteraceae | NDPG | 3.516202078 | 3.248687565 | <0.0001 |
| p__Bacteroidetes.c__Flavobacteriia | NDPG | 2.315423525 | 2.313257111 | 0.012397 |
| p__Bacteroidetes.c__Bacteroidia.o__Bacteroidales.f__Marinilabiaceae | NDPG | 2.128737194 | 2.84325491 | 0.006287 |
| p__Actinobacteria.c__Actinobacteria.o__Coriobacteriales | SGPG | 3.090723599 | 2.753276211 | 0.034721 |
| p__Fibrobacteres.c__Fibrobacteria | NDPG | 3.516202078 | 3.248687628 | <0.0001 |
| p__Firmicutes.c__Clostridia.o__Thermoanaerobacterales | NDPG | 2.298218262 | 2.569880682 | 0.011232 |
| p__Proteobacteria.c__Gammaproteobacteria.o__Xanthomonadales.f__norank_o__Xanthomonadales | HRGPG | 2.296161598 | 2.62995295 | 0.032644 |
| p__Fibrobacteres.c__Fibrobacteria.o__Fibrobacterales | NDPG | 3.516202078 | 3.248687019 | <0.0001 |
| p__Proteobacteria.c__Gammaproteobacteria.o__Pseudomonadales.f__Moraxellaceae | SGPG | 0.93134657 | 3.538836416 | 0.031221 |
| p__Elusimicrobia.c__Elusimicrobia | NDPG | 3.229921388 | 2.82815933 | 0.007238 |
| p__Actinobacteria.c__Actinobacteria | SGPG | 4.800212952 | 4.513129653 | <0.0001 |
| p__Cyanobacteria | NDPG | 4.123821232 | 3.737539353 | 0.011882 |
| p__Firmicutes.c__Negativicutes.o__Selenomonadales.f__Veillonellaceae | SGPG | 1.643468582 | 3.300418421 | 0.014726 |
| p__Lentisphaerae.c__Lentisphaeria.o__Victivallales.f__Victivallaceae | NDPG | 3.167998646 | 2.6954165 | 0.017289 |
| p__Firmicutes.c__Bacilli.o__Bacillales.f__Bacillaceae | SGPG | 3.419409985 | 3.139081422 | <0.0001 |
| p__Bacteroidetes.c__Bacteroidia.o__Bacteroidales.f__Bacteroidales_UCG-001 | NDPG | 2.710853534 | 2.607883081 | 0.001361 |
| p__Actinobacteria.c__Actinobacteria.o__Frankiales | SGPG | 2.600627196 | 2.509660134 | 0.000428 |
| p__Actinobacteria.c__Actinobacteria.o__Micrococcales.f__Micrococcaceae | SGPG | 4.777063883 | 4.494623549 | <0.0001 |
| p__Actinobacteria.c__Actinobacteria.o__Propionibacteriales.f__Propionibacteriaceae | HRGPG | 2.360561965 | 2.662633919 | 0.004756 |
| p__Proteobacteria.c__Deltaproteobacteria.o__Myxococcales.f__unclassified_o__Myxococcales | NDPG | 2.204289486 | 2.831384087 | 0.000156 |
| p__Actinobacteria.c__Actinobacteria.o__Corynebacteriales.f__Nocardiaceae | SGPG | 2.317741705 | 2.45771216 | 0.01654 |
| p__Cyanobacteria.c__Cyanobacteria.o__SubsectionIII.f__FamilyI_o__SubsectionIII | SGPG | 2.085712836 | 2.442155117 | 0.033299 |
| p__Proteobacteria.c__Alphaproteobacteria.o__Rhodospirillales.f__Acetobacteraceae | SGPG | 1.300539829 | 3.212308234 | 0.027534 |
| p__Bacteroidetes.c__Bacteroidia.o__Bacteroidales.f__Porphyromonadaceae | SGPG | 4.04690658 | 3.382882611 | 0.04361 |
| p__Chloroflexi.c__KD4-96.o__norank_c__KD4-96.f__norank_c__KD4-96 | HRGPG | 2.236715892 | 2.820106196 | 0.032644 |
| p__Cyanobacteria.c__Cyanobacteria.o__norank_c__Cyanobacteria.f__norank_c__Cyanobacteria | SGPG | 1.879350553 | 2.85093655 | 0.001478 |
| p__Saccharibacteria.c__Unknown_Class_p__Saccharibacteria | NDPG | 3.922668461 | 3.227742665 | 0.024729 |
| p__Chloroflexi.c__KD4-96.o__norank_c__KD4-96 | HRGPG | 2.236715892 | 2.746032485 | 0.032644 |
| p__Fibrobacteres | NDPG | 3.516202078 | 3.248687539 | <0.0001 |
| p__Actinobacteria.c__Actinobacteria.o__Corynebacteriales | SGPG | 2.317741705 | 2.466351668 | 0.01654 |
| p__Firmicutes.c__Clostridia.o__Thermoanaerobacterales.f__Thermoanaerobacteraceae | NDPG | 2.298218262 | 2.569942456 | 0.011232 |
| p__Verrucomicrobia.c__WCHB1-41.o__norank_c__WCHB1-41 | NDPG | 2.67924679 | 2.459050293 | 0.00744 |
| p__Cyanobacteria.c__Cyanobacteria.o__SubsectionIII | SGPG | 2.085712836 | 2.445315758 | 0.033299 |
| p__Cyanobacteria.c__Cyanobacteria.o__Gastranaerophilales | NDPG | 4.121310375 | 3.741961866 | 0.007318 |
| p__Bacteroidetes.c__Bacteroidia.o__Bacteroidales.f__Bacteroidales_BS11_gut_group | HRGPG | 4.246491124 | 3.7805521 | 0.01971 |
| p__Actinobacteria.c__Actinobacteria.o__Coriobacteriales.f__Coriobacteriaceae | SGPG | 3.090723599 | 2.753276211 | 0.034721 |
| p__Verrucomicrobia.c__Verrucomicrobiae.o__Verrucomicrobiales.f__Verrucomicrobiaceae | NDPG | 4.40195442 | 4.009110372 | 0.002613 |
| p__Elusimicrobia.c__Elusimicrobia.o__Elusimicrobiales.f__Elusimicrobiaceae | NDPG | 3.217890745 | 2.807067144 | 0.007238 |
| p__Elusimicrobia | NDPG | 3.229921388 | 2.82815933 | 0.007238 |
| p__Cyanobacteria.c__Cyanobacteria.o__Gastranaerophilales.f__norank_o__Gastranaerophilales | NDPG | 4.121310375 | 3.741961866 | 0.007318 |
| p__Verrucomicrobia.c__Verrucomicrobiae.o__Verrucomicrobiales | NDPG | 4.40195442 | 4.009110372 | 0.002613 |
| p__Proteobacteria.c__Alphaproteobacteria.o__Rhodospirillales | NDPG | 3.453320025 | 3.034323521 | 0.018024 |
| p__Elusimicrobia.c__Elusimicrobia.o__Elusimicrobiales | NDPG | 3.217890745 | 2.807067144 | 0.007238 |
| p__Proteobacteria.c__Alphaproteobacteria.o__Rhizobiales.f__unclassified_o__Rhizobiales | SGPG | 1.60713115 | 2.631853284 | 0.035549 |
| p__Actinobacteria.c__Actinobacteria.o__Micrococcales.f__Microbacteriaceae | SGPG | 2.733514318 | 2.53199338 | 0.005276 |
| p__Actinobacteria.c__Actinobacteria.o__Rubrobacterales | HRGPG | 2.441418432 | 2.507726631 | 0.009153 |
| p__Cyanobacteria.c__Cyanobacteria | NDPG | 4.123821232 | 3.737539353 | 0.011882 |
| p__Actinobacteria.c__Actinobacteria.o__Rubrobacterales.f__Rubrobacteriaceae | HRGPG | 2.441418432 | 2.51096482 | 0.009153 |
| p__Actinobacteria.c__Actinobacteria.o__Acidimicrobiales.f__Acidimicrobiaceae | HRGPG | 1.913105081 | 2.860724395 | 0.032644 |
| p__Bacteroidetes.c__Flavobacteriia.o__Flavobacteriales.f__Flavobacteriaceae | NDPG | 2.315423525 | 2.315531159 | 0.012397 |
| p__Chloroflexi | HRGPG | 3.135618984 | 2.876076202 | 0.041001 |
| p__Verrucomicrobia.c__WCHB1-41 | NDPG | 2.67924679 | 2.458935291 | 0.00744 |
| p__Actinobacteria.c__Actinobacteria.o__Acidimicrobiales | HRGPG | 1.913105081 | 2.802220739 | 0.032644 |
| p__Actinobacteria.c__Actinobacteria.o__Pseudonocardiales.f__Pseudonocardiaceae | SGPG | 1.378896072 | 3.033732769 | 0.029403 |
